# Oscillatory brain activity during multisensory attention reflects activation, disinhibition, and cognitive control

**DOI:** 10.1038/srep32775

**Published:** 2016-09-08

**Authors:** Uwe Friese, Jonathan Daume, Florian Göschl, Peter König, Peng Wang, Andreas K. Engel

**Affiliations:** 1 University Medical Center Hamburg-Eppendorf, Department of Neurophysiology and Pathophysiology, Hamburg, Germany; 2 Institute of Cognitive Science, University of Osnabrück, Germany

## Abstract

In this study, we used a novel multisensory attention paradigm to investigate attention-modulated cortical oscillations over a wide range of frequencies using magnetencephalography in healthy human participants. By employing a task that required the evaluation of the congruence of audio-visual stimuli, we promoted the formation of widespread cortical networks including early sensory cortices as well as regions associated with cognitive control. We found that attention led to increased high-frequency gamma-band activity and decreased lower frequency theta-, alpha-, and beta-band activity in early sensory cortex areas. Moreover, alpha-band coherence decreased in visual cortex. Frontal cortex was found to exert attentional control through increased low-frequency phase synchronisation. Crossmodal congruence modulated beta-band coherence in mid-cingulate and superior temporal cortex. Together, these results offer an integrative view on the concurrence of oscillations at different frequencies during multisensory attention.

The ability to direct attention voluntarily to specific aspects of our environment is essential for flexible, goal-directed behavior. Considerable progress in identifying relevant attention networks in the brain has been made in the last decades, and research has started to work out the underlying functional mechanisms by which these networks are formed and how they operate^1^,^2^. One promising candidate mechanism is the synchronisation of neuronal activity in distributed networks^3^,^4^. Such synchronisation can occur with diverse dynamics, and it has been proposed that oscillations in high- and low-frequency ranges might index different cortical operations in local and large-scale networks^5^. In the present study, we were specifically interested to relate activation, inhibition, and cognitive control processes to concurrent changes of high- and low-frequency oscillatory brain activity during a multisensory attention task. Since these processes are ubiquitous also in other cognitive domains, specifying their relationship to different brain rhythms contributes to a general understanding of how oscillatory activity subserves cognition.

Multisensory processing is particularly well suited to study the formation and function of brain networks because the informational entry points into the processing system are clearly segregated and their locations are well known. For tasks with stimulation in more than one modality, the different sensory cortices involved have to be activated and integrated into a coherent network. Moreover, it is reasonable to assume the participation of additional brain regions which – depending on the task – govern multisensory integration and exert top-down control functions^6^,^7^. While several studies have investigated synchronised neuronal oscillations during multisensory processing^8^, evidence for the influence of top-down attention in this context is still very sparse. Spatial attention was shown to modulate early evoked gamma-band power in response to audio-visual stimuli^9^. In a tactile discrimination task, Kanayama *et al.*^10^ varied the ratio of congruent and incongruent visual-tactile stimuli and demonstrated that top-down expectancy had an influence on oscillatory power in the theta and gamma range^10^. Göschl *et al.*^11^ employed a visual-tactile pattern matching task and found task-specific spectral signatures for theta-, alpha-, and beta-power depending on the cognitive demands^11^. Functional coupling analyses on a subset of their data revealed that large-scale synchronisations in the beta range might promote multisensory integration^12^. Taken together, the study of multisensory attention can provide insights about general brain mechanisms of local activation/inhibition as well as inter-regional communication and top-down control.

To further specify these mechanisms, we developed an experimental paradigm in which visual and auditory stimuli underwent two consecutive brief and weak changes in intensity (Fig. 1). Attention was cued to one of the two changes, and participants had to indicate whether the visual and auditory stimuli changed in a congruent or incongruent manner. Comparing cued and uncued intervals, we predicted that the allocation of attention would be associated with an activation of task relevant brain regions including early visual and auditory sensory cortices. Based on observations of typical signatures of attentional modulation in unisensory processing, we expected this activation to be reflected by an increase of gamma-band activity accompanied by decreased alpha-band activity^13^,^14^,^15^. Top-down control was supposed to be associated with increased frontal theta-band oscillations as well as increased low frequency phase coupling^16^. Finally, since previous studies have associated beta-band oscillations with attentional processes^17^ and the evaluation of crossmodal congruence^11^, we also assumed to find modulated beta-band activity.

## Results

For each participant, individual intensity levels for each change were determined preceding the experiment. Across participants, Michaelson contrast values during the increase of the visual stimuli ranged from 83.0% (15.1/1.4 cd/m^2^) to 98.5% (27.2/0.2 cd/m^2^), and contrast values during the decrease epochs ranged from 37.1% (9.8/4.5 cd/m^2^) to 7.0% (8.4/7.3 cd/m^2^), respectively. For the auditory stimuli, volume values during increases ranged from 81.4 dB to 87.7 dB, and volume values during decrease epochs ranged from 78.9 dB to 68.4 dB.

Data of two participants were discarded because of chance level performance or technical problems. The behavioral data of the remaining 19 participants revealed an overall mean accuracy of 83.1 ± 9.1% (mean ± s.d.) for the first change. Accuracy was significantly higher for congruent (85.4 ± 7.5) than for incongruent (80.7 ± 9.6) first changes, *t*_18_ = 2.71; *p* < 0.05.

### Spectral power

We selected frequency bands based on visual inspection of the grand average time-frequency representations as follows: 2.5–5 Hz delta/theta, 7.5–10 Hz alpha, 15–25 Hz beta, 60–80 Hz gamma. Figure 2 depicts spectral power averaged across posterior sensors with respect to the stimulus onset time window as well as the change time window. The low frequency band in the delta/theta range was defined based on time-frequency representations from frontal sensors not shown here (but see the topographical distributions spectral power in Fig. 3). For briefness we refer to this low frequency range as theta in the following (for a critical discussion of the common delta/theta distinction, see Lega *et al.*^18^). For statistical comparisons in source space we averaged power across all time bins in each time window separately. We restricted the stimulus onset and the change onset time windows to 0–500 ms to capture primarily activity associated with early stimulus processing and to avoid potential overlap with the respective subsequent intensity change. (Depending on the jitter interval, the change onset could follow the stimulus offset after 450 ms, and the second intensity change could follow the offset of the first change also after 450 ms).

Figure 2 reveals that the overall activity pattern across frequencies was very similar for stimulus onset and change onset. At stimulus onset, there was a broadband power increase for higher frequencies that became more defined for the change onset. For frequencies below 40 Hz, there was a strong decrease over posterior regions at stimulus onset that was pronounced after the change. The left panel of Fig. 3 illustrates the changes in spectral power in the stimulus onset time window with more topographical specificity based on contrasting the mean of all conditions against baseline activity. Gamma power increased significantly in occipital, parietal, posterior temporal, and posterior frontal cortex regions. Beta and alpha power decreased in almost the entire cortex – except for the most anterior frontal parts. Theta power decreased in occipital cortex, posterior inferior temporal cortex, and parietal cortex. There was also a frontal theta power increase in sensor space that did not surpass the FDR-corrected threshold in the source level analysis.

The statistical analyses of the change onset window revealed neither a significant effect for the factor congruence nor for the interaction between congruence and attention. As the right panel of Fig. 3 shows, attention modulated spectral power in all frequency bands studied. Overall, effects were located in regions that were also associated with the stimulus onset. In the gamma-band, attended versus unattended changes were associated with increased power in occipital, inferior and superior temporal, parietal and frontal cortex. Beta- and alpha-band decreases showed large topographical overlap and were located in widespread areas of the occipital, inferior temporal and posterior parietal cortex. In contrast to the window after stimulus onset, activity in frontal and anterior temporal regions was not modulated significantly in the change onset time window. In the theta-band, spectral power was decreased for attended versus unattended trials in occipital, superior parietal and temporal cortex, while there was an increase in theta power in medial frontal cortex.

To specifically investigate the influence of attention in early visual and auditory brain areas, we conducted a region of interest analysis (Fig. 4). In both, the visual and the auditory cortex there was a significant increase of gamma power and a decrease in theta, alpha, and beta power associated with the allocation of attention (visual cortex: theta *p* < 0.001, *p*_crit_ = 0.006; alpha *p* < 0.001, *p*_crit_ = 0.008; beta *p* < 0.001, *p*_crit_ = 0.007; gamma *p* = 0.005, *p*_crit_ = 0.01; auditory cortex: theta *p* = 0.003, *p*_crit_ = 0.015; alpha *p* = 0.006, *p*_crit_ = 0.05; beta *p* = 0.005, *p*_crit_ = 0.025; gamma *p* < 0.001, *p*_crit_ = 0.01).

### Source space coherence

In the analysis of source coherence, three significant clusters were identified in the time window corresponding to the first intensity change (Fig. 5). These clusters represent regions in which the experimental conditions modulated coherence in a specific frequency range. Crossmodal congruence modulated coherence in the beta-band. Attention was associated with two clusters in the alpha- and theta-band respectively. No significant clusters were found for the interaction between attention and congruence. The clustering approach resulted in a pooling of frequencies slightly different from the frequency bands predefined for the analysis of spectral power. The beta-band cluster arose in a narrow 30-32 Hz range in regions including left medial parietal cortex as well as right superior and middle temporal gyrus. These regions exhibited stronger coherence for the incongruent condition compared to the congruent condition in the beta-band. One of the two clusters associated with changes in attention concentrated coherence between 8–12 Hz in widespread posterior regions in occipital, parietal and temporal cortex. Within this cluster, coherence decreased for the attended condition compared to the unattended condition. Moreover, the allocation of attention was found to increase theta-band coherence in a cluster comprising bilateral inferior frontal and precentral gyrus, left middle temporal gyrus, and right medial frontal cortex. These clusters, identified with coherence analysis, could not be explained by volume conduction effects (see bar plots on the right hand side of Fig. 5). Average imaginary part of coherency, which reflects non-zero phase-lag synchronisation and is therefore not influenced by volume conduction, was significantly different between conditions within the clusters (beta congruence effect: *t*_18_ = −2.70, *p* = 0.015; alpha attention effect: *t*_18_ = −4.10, *p* < 0.001; theta attention effect: *t*_18_ = 3.62, *p* = 0.002). These effects mirror the significant differences found for coherence (beta congruence effect: *t*_18_ = −8.56, *p* < 0.001; alpha attention effect: *t*_18_ = −9.00, *p* < 0.001; theta attention effect: *t*_18_ = 8.75, *p* < 0.001). This strongly suggests that volume conduction did not contaminate the significant coherence cluster results.

## Discussion

We found focussing attention to combined audio-visual stimuli was accompanied by (i) augmented gamma power in a network including auditory and visual cortex, (ii) decreased alpha power and coherence in posterior, parietal and temporal regions, (iii) increased frontomedial theta power as well as increased theta coherence in frontal and temporal cortex. Task-relevant changes in audio-visual stimulus congruence were not associated with specific power differences, but with higher beta-band coherence for incongruent stimuli in medial parietal and temporal cortex. These results integrate findings from unimodal studies on changes in neural dynamics associated with attention^13^,^17^. Overall, our findings support the view that gamma oscillations reflect active processing in attention networks while alpha oscillations reflect inhibitory processes, and that frontal theta-band activity possibly relates to top-down control^16^.

Directing attention has been associated with increased cortical oscillatory activity in the gamma frequency range in previous studies^13^,^19^. Enhanced gamma-band responses for attended compared to unattended unisensory stimuli over the respective early sensory cortex regions have been observed for visual^17^,^20^, auditory^21^, and somatosensory stimuli^22^. In an MEG study with audio-visual speech stimuli, Kaiser *et al.*^23^ found simultaneous attention-dependent modulations in auditory and visual cortex^23^. In contrast, Kahlbrock *et al.*^24^ presented visual gratings accompanied by sound stimuli and found modulated gamma-band responses only in visual cortex^24^. Our results show enhancement of gamma-band power associated with the allocation of multisensory attention in a network comprising visual and auditory cortex as well as parietal, temporal and frontal regions. Rouhinen *et al.*^25^ identified a similar gamma network manipulating attentional load^25^. These findings support the view that gamma-band responses reflect activation in distributed attention networks. Importantly, we show for the audio-visual congruence evaluation task, top-down influence of multisensory attention extends to both early auditory and visual cortex.

In our study, the allocation of attention was accompanied by parallel decreases of alpha- and beta-band power in parietal, occipital and temporal cortex regions (Figs 3 and 4). The networks involved were strikingly similar, and it would be a matter of further research to establish in how far this is due to frequency smoothing or overlapping functional roles of these frequency bands. While attention has been implicated to modulate beta-band power^17^,^25^, more evidence and clearer theoretical grounds have been established for a close relationship between attention and alpha-band oscillations. An influential view holds that alpha activity inhibits task-irrelevant regions (“gating by inhibition”) – while gamma activity reflects active processing^15^. Decreased alpha activity in task-relevant regions is correspondingly interpreted as a release from inhibition. Our results are in close agreement with this latter notion. The spatial distributions of gamma power increases and alpha decreases were largely overlapping (Fig. 3), and the region of interest analysis confirmed this pattern for the visual and auditory cortices (Fig. 4). While concomitant parieto-occipital alpha decrease and gamma increase have been observed frequently in visual processing^26^,^27^,^28^, similar evidence for auditory processing is sparse^29^,^30^. Two recent studies found attention-dependent modulation of alpha band activity in auditory and visual cortex – but unfortunately these reports did not cover higher frequency oscillations^31^,^32^. In a study using audio-visual speech stimuli, Lange *et al.*^33^ found low frequency power to be decreased for congruent stimuli in auditory cortex, while high gamma power increased in frontal regions^33^. Moreover, the predictiveness of visual stimuli for task-irrelevant auditory stimuli has been shown to modulate alpha- and gamma-band activity in auditory cortex^34^. Our results provide strong support for the “gating by inhibition” hypothesis because we observed increased gamma and decreased alpha power in pre-defined early sensory regions. In addition, alpha-band coherence in occipital and parietal cortex was also found to be modulated by attention (Fig. 5). However, since coherence is based on combined phase/amplitude information, and we observed significant power modulations in the same brain areas, this finding cannot be interpreted unequivocally as a change of phase synchrony.

There is ample evidence that medial and lateral frontal cortex are associated with attention and cognitive control^1^. Low frequency oscillations have been proposed as a mechanism by which cognitive control functions could be implemented neurophysiologically^35^. This prospect rests on the idea that – depending on the phase of the low frequency oscillation – windows of high and low local excitability emerge^36^. Moreover, since low frequency oscillations can be assumed to be less prone to conduction delays, they are better suited to mediate long-range communication than high frequencies^37^. We found evidence for attention-dependent modulations of frontal theta oscillations in the analyses of spectral power and coherence. Spectral power was higher for the attended compared to the unattended condition in mediofrontal and lateral prefrontal cortex. The medial region was slightly anterior to the mid-cingular, pre-SMA region that has often been associated with frontal midline theta activity^35^. It is unclear whether this discrepancy arose because of imprecisions of MEG source localisation. On the other hand, the medial prefrontal cortex is a highly connected region, and its precise functional subdivisions are not completely resolved^38^. The source-space clustering approach revealed elevated coherence in bilateral inferior frontal and left temporal cortex for attended versus unattended audio-visual stimuli. Since these regions did not show increased spectral power, it is reasonable to assume that the larger phase-synchronisation under attention represents a non-trivial effect. To the best of our knowledge, this is the first report of modulated frontal theta coherence in the context of multisensory attention. By requiring the participants to evaluate weak intensity changes in two modalities simultaneously, the task imposes the need for strong attentional control. Recent behavioral studies have shown that vision, audition, and also haptics are likely to share attentional resources^39^,^40^. Our results indicate that frontal cortex theta-band activity might play a role in controlling the allocation of these resources. An alternative interpretation could relate the increase in theta synchronisation in the attended condition to memory encoding of the audio-visual stimuli or the respective response^41^. We regard it as more likely that frontal theta in our study is associated with the top-down control of attention since the task does not require long-term storage of the stimulus, and because the memory requirements to store the response are minimal.

A significant beta-band cluster in medial parietal cortex and right superior temporal gyrus exhibited larger coherence for incongruent compared to congruent stimuli. Beta-band activity has been linked to rather diverse cognitive processes including decision making and general integrative functions^42^ as well as the maintenance of the current sensorimotor or cognitive state^43^. With respect to crossmodal congruence, interactions between superior temporal sulcus and auditory cortex have been observed in beta-band coupling during audio-visual stimulation in macaque monkeys^44^. In humans, beta-band power in superior temporal cortex has been modulated by auditory noise during audiovisual speech processing^45^. Based on our behavioral results, it may be speculated that the difference in beta-band coherence might be related to incongruent trials being overall more difficult than congruent trials. Possibly, incongruent audio-visual changes engaged the mid-cingular region more because of its function in error-monitoring and decision making. The right temporal area that was also connected to a higher degree in incongruent trials is close to the auditory cortex. More difficult incongruent stimuli might have increased the need for communication between higher processing regions and early auditory areas.

Except for the aforementioned beta-band coherence cluster, congruent and incongruent stimuli elicited no differential oscillatory activity in our study. Given that we only analysed activity within 500 ms after change onset, this negative finding concurs with the results of Göschl *et al.*^11^ who found visual-tactile congruence effects building up only after 400 ms to 1100 ms^11^. (Unfortunately, we could not analyse this later time window in our data because it overlaps with the presentation of the second intensity change.) In typical studies demonstrating early multisensory congruence effects, mostly detection or categorisation tasks are employed in which cross-modal congruence per se is task-irrelevant^10^,^46^. Overall, the results of Göschl *et al.*^11^ and from our study indicate that if cross-modal congruence is task-relevant, early multisensory congruence effects seem to be diminished.

In conclusion, we found that directing attention to bimodal changes in the audio-visual stimulus stream was associated with a coherent pattern of high- and low-frequency oscillations. Presumably, spectral power in gamma- and alpha-band reflected activation and inhibitory processes, respectively, whereas frontal theta-band coherence was linked to cognitive control. These results contribute to an integrative account on how cognitive functions are implemented neurophysiologically by rhythmic brain activity.

## Methods

### Participants

Twenty-one healthy volunteers participated in the experiment (7 female; mean ± s.d. age: 26.4 ± 4.0 years; all right-handed) and received monetary compensation for their participation. All participants reported normal or corrected-to-normal vision, normal hearing and no history of neurological or psychiatric illness. The ethics committee of the Medical Association Hamburg approved the study. Informed consent was obtained from all participants prior to each recording, and the experiment was carried out in accordance with the approved guidelines and regulations.

### Stimuli and procedure

**Figure 1:**
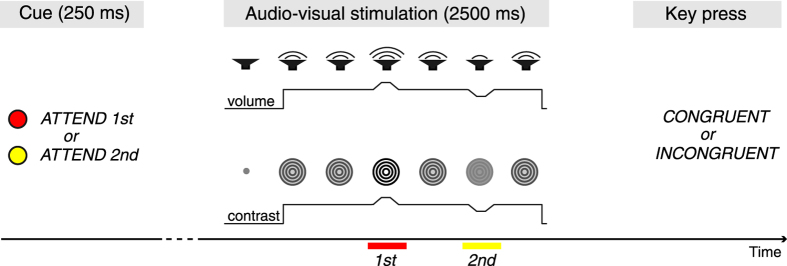
Schematic illustration of the trial sequence. First, in each trial, a color cue informed participants to direct attention to one of two upcoming brief intensity changes during the audio-visual stimulation. Next, a complex sound and a circular drifting grating were presented. Two simultaneous 300 ms changes in sound volume and visual contrast occurred during the 2500 ms stimulation. After stimulation offset, participants indicated whether for the attended interval the two modalities changed in the same direction (congruent) or in different directions (incongruent). In this example both first and second changes are congruent. In the experiment increases and decreases in both modalities varied independently.

**Figure 2:**
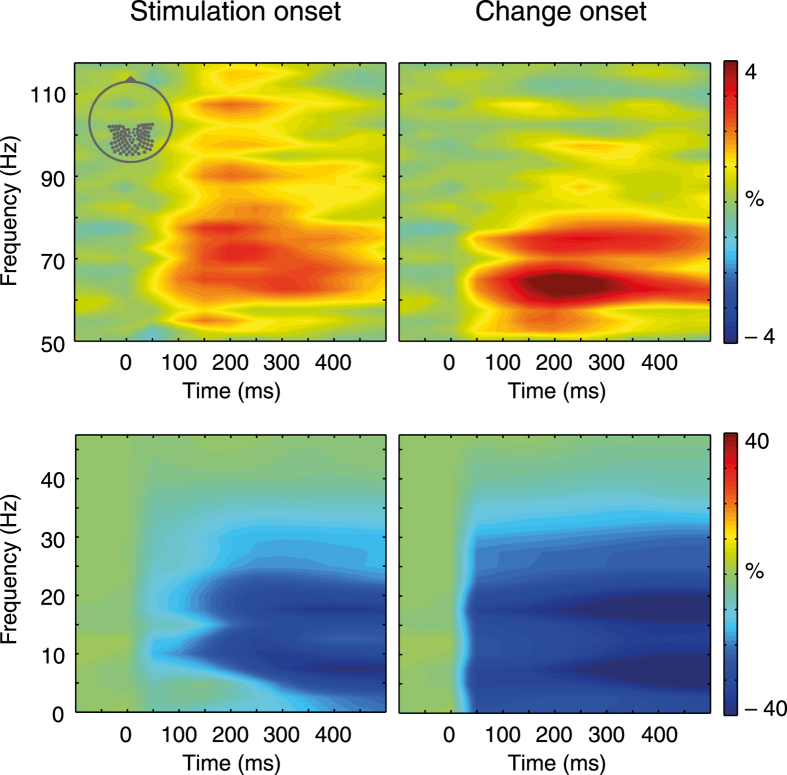
Grand mean time-frequency representations of spectral power corresponding to the stimulus onset time window (left) and the change onset window (right) for posterior sensor sites (inset at the upper left). The frequency range was split such that higher frequencies are illustrated in the upper two panels while the lower frequencies are shown below. Oscillatory power was calculated across conditions relative to the pre-cue baseline. Colors represent percentage signal change with respect to pre-cue baseline activity.

**Figure 3:**
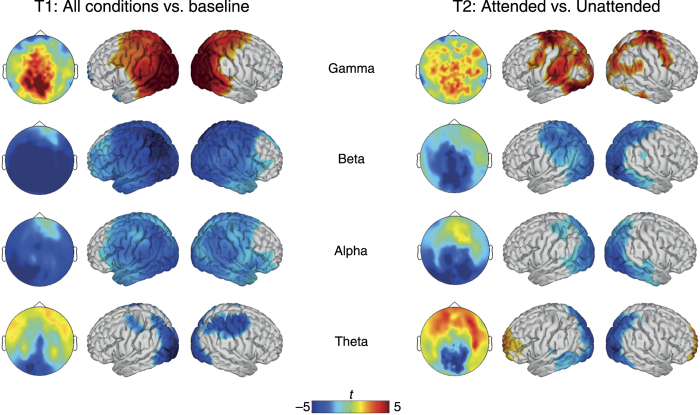
Differences in oscillatory power with respect to all conditions versus baseline at stimulus onset (T1) on the left, and the effect of attention at change onset (T2) on the right. Topographical maps depict the sensor space distribution of *t*-values. For the corresponding source level effects, colors indicate *t*-values with non-significant values set to zero (FDR corrected).

**Figure 4:**
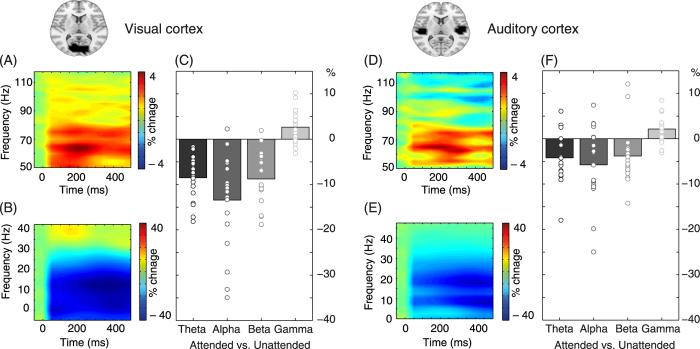
Spectral power changes relative to the pre-cue baseline in visual cortex on the left and auditory cortex on the right. Grand average time-frequency representations of spectral power corresponding to the change onset time window were averaged across conditions and all grid points within the respective region of interest (depicted as dark areas on horizontal MRI slices). Overall, the pattern of source-level spectral changes in the sensory cortices closely correspond to the global sensor-level changes shown in Fig. 2. (A,D) Time-frequency representations of spectral power for higher frequencies. (**B**,**E**) Time-frequency representations of spectral power for lower frequencies. (**C**,**F**) Bar plots illustrating the differential effect of attention for each frequency band. Bars represent grand means, and circles represent data from each participant to illustrate interindividual variance. All mean deviations from zero are significant. At the group level, spectral power in theta-, alpha-, and beta-band decreased while gamma-band power increased in both visual and auditory cortex.

**Figure 5:**
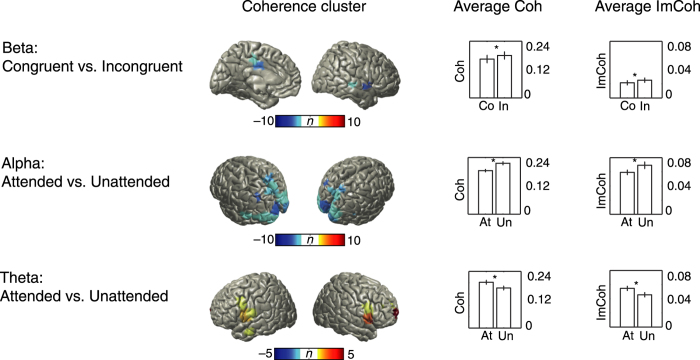
Clusters with significant changes in source coherence were found for the congruence effect in the beta-band (upper row), as well as for the attention effect in the alpha-band (middle row) and theta-band (bottom row). Each significant coherence cluster is illustrated with colors indicating the number of connections of a given location to other locations within the cluster (see methods for details). For the bar plots on the right hand side of the figure coherence and imaginary part of coherency were calculated between all voxels constituting a significant cluster. Bars represent average coherence or average imaginary part of coherency, respectively. Error bars denote standard errors, and asterisks indicate significant differences. Since all condition differences of average imaginary part of coherency are significant, it is unlikely that the source cluster coherence results are compromised by volume conduction. *Abbreviations*: Coh – coherence, ImCoh – imaginary part of coherency, Co – congruent, In – incongruent, At – attended, Un – unattended.

Each trial began with a light-gray fixation dot (size: 0.3° visual angle) at the center of the screen presented against a dark gray background. After 1500 to 2000 ms (jittered randomly in steps of 100 ms across trials) the fixation dot either turned red or yellow for a duration of 250 ms. The color cued attention to one of two consecutive feature changes of the following audio-visual stimulation. Subsequently, the grey fixation dot was again presented for 750 ms followed by a 2500 ms audio-visual stimulation. Figure 1 illustrates the sequence within a trial.

The visual stimulus consisted of a circular black-and-white sinusoidal grating (size: 5° visual angle; spatial frequency: 1.67° visual angle, Michelson contrast: 62.0%), which was drifting either inwards or outwards (speed: 1.25 Hz). The initial spatial phase of the grating was randomised and the movement direction was counterbalanced within each block. The auditory stimulus was a complex sinusoidal sound created by modulating high-frequency carrier signals (13 sine waves: 64 Hz and its first 6 harmonics as well as 91 Hz and its first 5 harmonics) with a low-frequency modulator (1.25 Hz). Subjectively, this stimulus sounded similar to a chord played by an organ. The average volume corresponded to 80 dB SPL.

Twice during the presentation of the audio-visual stimulus, short simultaneous changes in contrast and volume occurred for 300 ms. In detail, 750 to 1250 ms after stimulus onset the participants were presented with a first change, followed by a second change 1500 to 2000 ms after stimulus onset. The onset of each change was pseudo-randomly jittered in steps of 100 ms but the onset of the second change occurred at the earliest 750 ms after the first. During each change, both contrast and volume could either shortly increase or decrease independently. Contrast and volume were ramped up or down to the maximum or minimum value within 100 ms, respectively, kept their plateaus for further 100 ms and were ramped back to the original value within another 100 ms. The default contrast of the grating was 62% and the default volume of the sound was 80 dB. Change magnitudes were individually determined for each participant (see below).

In each trial, participants had to specify whether the cued change represented a congruent or incongruent change in volume and contrast. We instructed participants to respond as fast and accurately as possible as soon as the audio-visual stimulus ended by pressing one of two response buttons with their right index or middle finger, respectively. Response buttons were counterbalanced between participants. A grey plus or minus sign presented for 250 ms indicated whether the given response was correct or incorrect, respectively. Afterwards, participants initiated the next trial with another button press.

The experiment consisted of 512 trials divided into eight blocks of 64 trials. After each block a short break was scheduled. The experiment was split into two sessions with the second session being recorded on the next day at the earliest or after 21 days at the latest (mean ± s.d. time between sessions: 4.3 ± 4.7 days).

To minimise changes in oscillatory activity that would simply reflect intensity changes of the stimuli, we determined the magnitude of changes individually for each participants for both modalities and change directions. Our goal was to find the minimal intensity changes at which participants could perform the task with about 80% accuracy. An elaborate 6-step training procedure was required to reach this goal: (1) To familiarise the participants with the stimuli, example stimuli with highly above-threshold changes in volume or contrast were presented repeatedly. (2) Subsequently, participants determined preliminary thresholds in volume and contrast change by choosing the smallest detectable difference with a simple staircase procedure. These thresholds served as the initial guess for the Bayesian adaptive staircase procedure QUEST that would follow later^47^. (3) Next, a training phase was conducted which exactly represented the procedure of the main experiment, with the exception that again high-above threshold change intensities were used. As soon as participants reached at least 90% accuracy within the last 20 trials, the training was terminated. (4) Participants then performed uni- and bimodal versions of the QUEST procedure as follows. QUEST concentrates change perception near threshold by trial-wise updating an underlying estimated psychophysical function depending on a given response by the observer. For each modality we presented 60 trials in a randomly intermixed sequence, with half of the trials containing an increase and the other half a decrease in volume. Individual response patterns led to individual increment and decrement thresholds concentrated by two parallel QUEST procedures at 90% correct level. (5) The unimodally retrieved thresholds served as the initial guess for an additional bimodal version of the QUEST procedure containing the audio-visual stimulus with a single simultaneous change in volume and contrast. Again, for each modality 60 trials were presented. Four parallel QUEST procedures concentrated observations at 90% correct level by analysing individual perception reports given separately for the auditory and the visual modality after each trial. Participants always first responded to the volume, then to the contrast change. (6) Based on a pilot study, the thresholds of the bimodal QUEST procedure were multiplied by a factor of 1.5 and then served as the individual volume and contrast change amplitudes in the main experiment.

Stimuli were generated using Matlab (Version: 8.0, R2012b; MathWorks, Natick, MA) and Psychtoolbox^48^ on a Dell Precision T5500 with Windows 7 Professional 64-bit operating system. The visual stimuli were backprojected onto a screen mounted to the MEG dewar at 60 Hz with a resolution of 1280 × 1024 pixels positioned 65 cm in front of the participants. The auditory stimulus was presented using MEG-compatible in-ear headphones (STAX, SRM-2525).

### MEG recordings and data preprocessing

MEG was recorded at a sampling rate of 1200 Hz using a 275-channel whole-head system (Omega, CTF Systems Inc.). Five channels were malfunctioning and therefore discarded from analysis. Additional Ag/AgCl-electrodes were used to record the horizontal and vertical electrooculogram (H/V-EOG) and the electrocardiogram (ECG).

Physiological data analysis was performed in Matlab (MathWorks, Natick, MA) using the M/EEG analysis toolbox FieldTrip (http://www.ru.nl/fcdonders/fieldtrip) and custom code. All recorded data sets (i.e. sessions) were separately preprocessed and artifacts were rejected before further analysis. First, the data were cut into epochs of 4 s including −1.5 s to 2.5 s relative to stimulus onset. Trials containing jumps and strong muscle artifacts were detected by semi-automatic procedures implemented in FieldTrip and were rejected after visual inspection. On average, 493.0 ± 14.9 trials (mean ± s.d.) of the total of 512 trials remained after this step. Data were filtered with a high-pass filter at 1 Hz and a low-pass filter at 170 Hz. Additionally, line noise artifacts were removed using band-stop filters between 49 and 51, 99 and 101 and 149 and 151 Hz. Next, we down-sampled the data to 600 Hz and performed an independent component analysis (ICA) to manually remove remaining eye-movements, cardiac and muscle artifacts based on careful inspection of the component time course, spectrum and topography^49^. On average, 35.8 ± 15.7 components (mean ± s.d.) were rejected per session.

### Spectral analyses

We analysed oscillatory activity with two complementary approaches. First, we looked at spectral power derived from pre-selected time-frequency windows. Second, we analysed coherence in source space with a more data-driven approach that allows the identification of large-scale cortical networks in space and frequency.

To estimate spectral power for frequency and time at each sensor we defined three time windows of interest in each trial: (1) The baseline was represented by 300 ms preceding the onset of the attentional cue. (2) Activity in response to the basic stimulus features was analysed in a window from 0–500 ms with respect to the onset of the audio-visual stimulation. (3) Activity associated with the experimental manipulations was analysed in a window from 0–500 ms with respect to the onset of the first audio-visual intensity change. We did not evaluate the second audio-visual intensity change because we assumed that for this later epoch the contrast between attended and unattended conditions would be confounded with processes related to memory maintenance and response preparation. We derived oscillatory power by sliding a 400 ms window in steps of 50 ms across the respective time range. Within these ranges a Hanning windowed fast Fourier transform (FFT) was computed. We analysed frequencies between 2.5 and 117.5 Hz in steps of 2.5 Hz (i.e. 47 frequencies). To obtain robust estimates of spectral power for each participant, we averaged the individual cross-spectral densities at each frequency and time point across all trials within one experimental condition. To achieve comparable signal-to-noise ratios across conditions the number of trials averaged per condition was matched to the number of trials of the condition with lowest accuracy, and we restricted the analyses to trials with correct responses. The diagonal of each cross-spectral density matrix constituted the spectral power estimate at each sensor.

To estimate spectral power at the cortical source level, we used linear beamforming^50^,^51^. Computations were made using a 5003 voxel continuous grid based on individual MR images recorded from each participant. The grid was aligned to the MNI152 template brain (Montreal Neurological Institute, MNI; http://www.mni.mcgill.ca). Leadfields were calculated using the single-shell volume conductor model^52^. In order to compare the conditions statistically, we first computed common filters by averaging the cross-spectral density matrices across all conditions and time points. These matrices were then used to obtain three orthogonal spatial filters for all frequencies and spatial locations, which were further combined to a single filter pointing in the direction of maximal variance and weighted with the first eigenvector’s elements^53^. To estimate the spectral power for each frequency and time point at each source location, the real-valued common filters were then multiplied with each cross-spectral density matrix and the diagonal of the result again defined the spectral power for a given frequency and time point and each source location. This procedure was carried out separately for each recording session, and data were afterwards averaged in source space.

For statistical analyses of oscillatory power we defined time-frequency windows of interest based on visual inspection of time-frequency representations of power associated with *stimulus onset* and averaged across all conditions and participants. This selection is independent of the main contrast of interest in this study – i.e. the “attended versus unattended” comparison in the *change onset* time window. For the chosen time-frequency windows we computed *t*-tests in source space for the average of all conditions versus baseline to illustrate the activity in each frequency band associated with general processing of the audio-visual stimuli. Our experimental manipulations were evaluated with respect to change onset using *t*-tests representing the 2 (attention) x 2 (congruence) design. The levels of the factor attention were compared aggregating across the levels of the factor congruence and vice versa. The interaction was analysed by contrasting the difference between attended-congruent and unattended-congruent with the difference of attended-incongruent and unattended-incongruent conditions. For individual statistical tests we assumed a significance level of *p* = 0.05, and we controlled for multiple comparisons by setting the false-discovery rate to *q* = 0.05^54^. Furthermore, to specifically investigate the influence of attention in early visual and auditory brain areas we performed a region-of-interest analysis. We defined MNI center coordinates (based on the Anatomy toolbox^55^) for the primary visual cortex (−9, −89, 6; 14, −84, 6) and the primary auditory cortex (−47, −23, 13; 51, −18, 10), and averaged data corresponding to the 80^th^ percentile from all grid points within a 1.5 cm radius. This procedure was chosen to account for inter-individual anatomical differences and the fuzziness of MEG source reconstructions. (Radii between 1–2 cm and the use of the median as summary statistic resulted in comparable results.) Condition differences were then calculated using *t*-tests with family-wise error rate control according to Holm-Bonferroni.

### Coupling analyses

To analyse functional coupling between brain regions based on oscillatory phase information, we employed an approach proposed by Hipp *et al.*^53^. Since this method requires the pairwise calculation of coherence for each source location and time/frequency bin, we reduced the source space to 324 locations covering the cortical surface of both hemispheres in registration to the ICBM152 template brain^56^. Leadfield calculation was realised with a single-shell head model^52^, and we used the linearly constrained minimum variance (LCMV) beamforming method to estimate the source activation in each cortical source location. Spectral estimates corresponding to the change onset interval were computed across 21 logarithmically scaled frequencies from 4 to 128 Hz with 0.25 octave steps using the multitaper approach^57^. To yield a spectral smoothing of approximately 0.75 octave across the whole frequency range, we used a variable number of slepian tapers with a temporal window size of 300 ms for frequencies larger or equal to 16 Hz. For lower frequencies, we used a single slepian taper and adjusted the time window accordingly. We stratified the number of trials used for the comparison between conditions as described in the analysis of oscillatory power. Moreover, a nonlinear z-transformation was applied to render the distribution of coherence values approximately Gaussian before applying the clustering approach^58^.

The identification of networks was carried out as follows. Instead of analysing interactions between two cortical areas in a two-dimensional space (location by location), we considered a three-dimensional space by also including the frequency dimension. We first computed coherence for all pairs of locations in all frequency bins and conditions. Then *t*-tests were conducted across participants, representing the effects of attention, congruence, and the interaction. The resulting three-dimensional matrices (location by location by frequency) for each comparison were then binarised to be 1 (connected) or 0 (not connected) based on a *t*-value threshold equivalent to *p* < 0.01. A connection was determined by three values: one frequency and two locations (of a pair). For neighboring connections we defined that at least two of the three values had to be identical, and the third value must not differ by more than one step. In other words, connections were neighbors if they were adjoined in location or if they differed by 0.25 octaves in frequency. The neighborhood of source locations was defined during source space construction using topological relationships. To remove spurious connections we spatially filtered the result matrices. Instead of defining a fixed number of connections as filter criterion, we proceeded as follows. A connection was only considered if its neighboring connections exceeded 30% of all possible connections. That is, for a given connection between location A and location B at frequency F, the maximal number of connections *n*_max_ is given by the sum of: (1) connections between A and B at frequency F+1 or F-1, (2) connections between A’s neighbors and B at F, and (3) connections between B’s neighbors and A at F. If a given connection had less than 0.3 * *n*_max_ neighboring connections, it was removed. The numerical size of the threshold criterion primarily affects the cluster size, and it was set arbitrarily. Eventually, the remaining cluster of connections formed the candidates for statistics. The size of a cluster is defined as the integral of the *t*-scores across all its connections. The significance of clusters was tested with permutation statistics. The network-identification approach was repeated 2000 times with shuffled condition labels which yielded an distribution of cluster sizes under the assumption of no differences between conditions^59^. This null-distribution was constructed by pooling the largest cluster sizes (two-tailed) of each permutation. Only clusters with sizes ranked top 5% in these null distributions were considered as significant (i.e. *p* = 0.05). The final clusters corresponded to networks of cortical regions with different synchronisation status among comparisons, and were continuous across frequency and pairwise space. Applying this method, we compensated for the relatively arbitrary thresholding procedure during the generation of the 3-D connectivity array: the clusters with fewer statistically stronger connections and clusters with more statistically weaker connections were balanced. In fact, the results were similar when choosing a different threshold.

To visualise the results, we computed the integral of the connections over frequency and target locations for each source location in all significant clusters. This provides the spatial distribution of the networks. The result was then illustrated by interpolating the number of connections from 324 to ~300,000 vertices on a template brain pial surface. The brain area involved for each cluster was identified according to surface-based atlases^60^.

Lastly, we evaluated the impact of possible volume conduction effects on our coherence analysis. Because of volume conduction effects, two locations may show larger coherence values due to a common “real source”. If so, their coupling should be characterised by zero phase-lag. If the imaginary part of coherency – reflecting only non-zero phase-lag synchronisation – differs between two locations, this coupling cannot be explained by volume conduction^61^. For each condition, we averaged the absolute values of imaginary part of coherency across the connections within a cluster. These means were used as indicators for non-zero phase-lag synchronisation, and we conducted *t*-tests to evaluate differences between the conditions (with Bonferroni alpha-level correction to account for multiple comparisons). For comparison, we also computed analogous average coherence values.

## Additional Information

**How to cite this article**: Friese, U. *et al.* Oscillatory brain activity during multisensory attention reflects activation, disinhibition, and cognitive control. *Sci. Rep.* **6**, 32775; doi: 10.1038/srep32775 (2016).

## References

[b1] Petersen S. E. & Posner M. I. The attention system of the human brain: 20 years after. Annu Rev Neurosci. 35, 73–89 (2012). 22524787 10.1146/annurev-neuro-062111-150525 PMC3413263

[b2] Miller E. K. & Buschman T. J. Cortical circuits for the control of attention. Curr Opin Neurobiol. 23, 216–222 (2013). 23265963 10.1016/j.conb.2012.11.011 PMC3709832

[b3] Engel A. K., Fries P. & Singer W. Dynamic predictions: oscillations and synchrony in top-down processing. Nat Rev Neurosci. 2, 704–716 (2001). 11584308 10.1038/35094565

[b4] Womelsdorf T. *et al.* Modulation of neuronal interactions through neuronal synchronization. Science 316, 1609–1612 (2007). 17569862 10.1126/science.1139597

[b5] von Stein A. & Sarnthein J. Different frequencies for different scales of cortical integration: from local gamma to long range alpha/theta synchronization. Int J Psychophysiol. 38, 301–313 (2000). 11102669 10.1016/s0167-8760(00)00172-0

[b6] Talsma D., Senkowski D., Soto-Faraco S. & Woldorff M. G. The multifaceted interplay between attention and multisensory integration. Trends Cogn Sci. 14, 400–410 (2010). 20675182 10.1016/j.tics.2010.06.008 PMC3306770

[b7] Driver J. & Noesselt T. Multisensory interplay reveals crossmodal influences on ‘sensory-specific’ brain regions, neural responses, and judgments. Neuron 57, 11–23 (2008). 18184561 10.1016/j.neuron.2007.12.013 PMC2427054

[b8] Engel A. K., Senkowski D. & Schneider T. R. In The Neural Bases of Multisensory Processes (eds Murray M. M. & Wallace M. T.) 115–130 (CRC Press, Boca Raton (FL), 2012). 22593873

[b9] Senkowski D., Talsma D., Herrmann C. S. & Woldorff M. G. Multisensory processing and oscillatory gamma responses: effects of spatial selective attention. Exp Brain Res. 166, 411–426 (2005). 16151775 10.1007/s00221-005-2381-z

[b10] Kanayama N., Tame L., Ohira H. & Pavani F. Top down influence on visuo-tactile interaction modulates neural oscillatory responses. Neuroimage 59, 3406–3417 (2012). 22173297 10.1016/j.neuroimage.2011.11.076

[b11] Göschl F., Friese U., Daume J., König P. & Engel A. K. Oscillatory signatures of crossmodal congruence effects: An EEG investigation employing a visuotactile pattern matching paradigm. Neuroimage 116, 177–186 (2015). 25846580 10.1016/j.neuroimage.2015.03.067

[b12] Wang P., Göschl F., Friese U., König P. & Engel A. K. Large-scale cortical synchronization promotes multisensory processing: An EEG study of visuotactile pattern matching. (submitted).

[b13] Fries P. Neuronal gamma-band synchronization as a fundamental process in cortical computation. Annu Rev Neurosci. 32, 209–224 (2009). 19400723 10.1146/annurev.neuro.051508.135603

[b14] Siegel M., Donner T. H. & Engel A. K. Spectral fingerprints of large-scale neuronal interactions. Nat Rev Neurosci. 13, 121–134 (2012). 22233726 10.1038/nrn3137

[b15] Jensen O. & Mazaheri A. Shaping functional architecture by oscillatory alpha activity: gating by inhibition. Front Hum Neurosci. 4, 186 (2010). 21119777 10.3389/fnhum.2010.00186 PMC2990626

[b16] Clayton M. S., Yeung N. & Cohen Kadosh R. The roles of cortical oscillations in sustained attention. Trends Cogn Sci. 19, 188–195 (2015). 25765608 10.1016/j.tics.2015.02.004

[b17] Siegel M., Donner T. H., Oostenveld R., Fries P. & Engel A. K. Neuronal synchronization along the dorsal visual pathway reflects the focus of spatial attention. Neuron 60, 709–719 (2008). 19038226 10.1016/j.neuron.2008.09.010

[b18] Lega B. C., Jacobs J. & Kahana M. Human hippocampal theta oscillations and the formation of episodic memories. Hippocampus 22, 748–761 (2012). 21538660 10.1002/hipo.20937

[b19] Jensen O., Kaiser J. & Lachaux J. P. Human gamma-frequency oscillations associated with attention and memory. Trends Neurosci. 30, 317–324 (2007). 17499860 10.1016/j.tins.2007.05.001

[b20] Koelewijn L., Rich A. N., Muthukumaraswamy S. D. & Singh K. D. Spatial attention increases high-frequency gamma synchronisation in human medial visual cortex. Neuroimage 79, 295–303 (2013). 23651840 10.1016/j.neuroimage.2013.04.108

[b21] Ray S., Niebur E., Hsiao S. S., Sinai A. & Crone N. E. High-frequency gamma activity (80-150Hz) is increased in human cortex during selective attention. Clin Neurophysiol. 119, 116–133 (2008). 18037343 10.1016/j.clinph.2007.09.136 PMC2444052

[b22] Bauer M., Oostenveld R., Peeters M. & Fries P. Tactile spatial attention enhances gamma-band activity in somatosensory cortex and reduces low-frequency activity in parieto-occipital areas. J Neurosci. 26, 490–501 (2006). 16407546 10.1523/JNEUROSCI.5228-04.2006 PMC6674422

[b23] Kaiser J., Hertrich I., Ackermann H. & Lutzenberger W. Gamma-band activity over early sensory areas predicts detection of changes in audiovisual speech stimuli. Neuroimage 30, 1376–1382 (2006). 16364660 10.1016/j.neuroimage.2005.10.042

[b24] Kahlbrock N., Butz M., May E. S. & Schnitzler A. Sustained gamma band synchronization in early visual areas reflects the level of selective attention. Neuroimage 59, 673–681 (2012). 21784164 10.1016/j.neuroimage.2011.07.017

[b25] Rouhinen S., Panula J., Palva J. M. & Palva S. Load dependence of beta and gamma oscillations predicts individual capacity of visual attention. J Neurosci. 33, 19023–19033 (2013). 24285906 10.1523/JNEUROSCI.1666-13.2013 PMC6618707

[b26] Brookes M. J. *et al.* GLM-beamformer method demonstrates stationary field, alpha ERD and gamma ERS co-localisation with fMRI BOLD response in visual cortex. Neuroimage 26, 302–308 (2005). 15862231 10.1016/j.neuroimage.2005.01.050

[b27] Sauseng P. *et al.* Brain oscillatory substrates of visual short-term memory capacity. Curr Biol. 19, 1846–1852 (2009). 19913428 10.1016/j.cub.2009.08.062

[b28] Friese U., Supp G. G., Hipp J. F., Engel A. K. & Gruber T. Oscillatory MEG gamma band activity dissociates perceptual and conceptual aspects of visual object processing: a combined repetition/conceptual priming study. Neuroimage 59, 861–871 (2012). 21835246 10.1016/j.neuroimage.2011.07.073

[b29] Crone N. E., Boatman D., Gordon B. & Hao L. Induced electrocorticographic gamma activity during auditory perception. Brazier Award-winning article, 2001. Clin Neurophysiol. 112, 565–582 (2001). 11275528 10.1016/s1388-2457(00)00545-9

[b30] Edwards E., Soltani M., Deouell L. Y., Berger M. S. & Knight R. T. High gamma activity in response to deviant auditory stimuli recorded directly from human cortex. J Neurophysiol. 94, 4269–4280 (2005). 16093343 10.1152/jn.00324.2005

[b31] Mazaheri A. *et al.* Region-specific modulations in oscillatory alpha activity serve to facilitate processing in the visual and auditory modalities. Neuroimage 87, 356–362 (2014). 24188814 10.1016/j.neuroimage.2013.10.052

[b32] van Driel J., Knapen T., van Es D. M. & Cohen M. X. Interregional alpha-band synchrony supports temporal cross-modal integration. Neuroimage 101, 404–415 (2014). 25042447 10.1016/j.neuroimage.2014.07.022

[b33] Lange J., Christian N. & Schnitzler A. Audio-visual congruency alters power and coherence of oscillatory activity within and between cortical areas. Neuroimage 79, 111–120 (2013). 23644355 10.1016/j.neuroimage.2013.04.064

[b34] van Wassenhove V. & Grzeczkowski L. Visual-induced expectations modulate auditory cortical responses. Front Neurosci. 9, 11 (2015). 25705174 10.3389/fnins.2015.00011 PMC4319385

[b35] Cavanagh J. F. & Frank M. J. Frontal theta as a mechanism for cognitive control. Trends Cogn Sci. 18, 414–421 (2014). 24835663 10.1016/j.tics.2014.04.012 PMC4112145

[b36] Fries P. A mechanism for cognitive dynamics: neuronal communication through neuronal coherence. Trends Cogn Sci. 9, 474–480 (2005). 16150631 10.1016/j.tics.2005.08.011

[b37] von Stein A., Chiang C. & König P. Top-down processing mediated by interareal synchronization. Proc Natl Acad Sci USA 97, 14748–14753 (2000). 11121074 10.1073/pnas.97.26.14748 PMC18990

[b38] Euston D. R., Gruber A. J. & McNaughton B. L. The role of medial prefrontal cortex in memory and decision making. Neuron 76, 1057–1070 (2012). 23259943 10.1016/j.neuron.2012.12.002 PMC3562704

[b39] Wahn B. & König P. Vision and haptics share spatial attentional resources and visuotactile integration is not affected by high attentional load. Multisensory Research 28, 371–391 (2015). 26288905 10.1163/22134808-00002482

[b40] Wahn B. & König P. Audition and vision share spatial attentional resources, yet attentional load does not disrupt audiovisual integration. *Frontiers in Psychology* (in press). 10.3389/fpsyg.2015.01084 PMC4518141 26284008

[b41] Anderson K. L., Rajagovindan R., Ghacibeh G. A., Meador K. J. & Ding M. Theta oscillations mediate interaction between prefrontal cortex and medial temporal lobe in human memory. Cereb Cortex 20, 1604–1612 (2010). 19861635 10.1093/cercor/bhp223

[b42] Donner T. H. & Siegel M. A framework for local cortical oscillation patterns. Trends Cogn Sci. 15, 191–199 (2011). 21481630 10.1016/j.tics.2011.03.007

[b43] Engel A. K. & Fries P. Beta-band oscillations–signalling the status quo? Curr Opin Neurobiol 20, 156–165 (2010). 20359884 10.1016/j.conb.2010.02.015

[b44] Kayser C. & Logothetis N. K. Directed Interactions Between Auditory and Superior Temporal Cortices and their Role in Sensory Integration. Front Integr Neurosci. 3, 7 (2009). 19503750 10.3389/neuro.07.007.2009 PMC2691153

[b45] Schepers I. M., Schneider T. R., Hipp J. F., Engel A. K. & Senkowski D. Noise alters beta-band activity in superior temporal cortex during audiovisual speech processing. Neuroimage 70, 101–112 (2013). 23274182 10.1016/j.neuroimage.2012.11.066

[b46] Mishra J. & Gazzaley A. Attention distributed across sensory modalities enhances perceptual performance. J Neurosci. 32, 12294–12302 (2012). 22933811 10.1523/JNEUROSCI.0867-12.2012 PMC3449148

[b47] Watson A. B. & Pelli D. G. QUEST: a Bayesian adaptive psychometric method. Percept Psychophys 33, 113–120 (1983). 6844102 10.3758/bf03202828

[b48] Brainard D. H. The Psychophysics Toolbox. Spat Vis 10, 433–436 (1997). 9176952

[b49] Hipp J. F. & Siegel M. Dissociating neuronal gamma-band activity from cranial and ocular muscle activity in EEG. Front Hum Neurosci. 7, 338 (2013). 23847508 10.3389/fnhum.2013.00338 PMC3706727

[b50] Van Veen B. D., van Drongelen W., Yuchtman M. & Suzuki A. Localization of brain electrical activity via linearly constrained minimum variance spatial filtering. IEEE Trans Biomed Eng. 44, 867–880 (1997). 9282479 10.1109/10.623056

[b51] Gross J. *et al.* Dynamic imaging of coherent sources: Studying neural interactions in the human brain. Proc Natl Acad Sci USA 98, 694–699 (2001). 11209067 10.1073/pnas.98.2.694 PMC14650

[b52] Nolte G. The magnetic lead field theorem in the quasi-static approximation and its use for magnetoencephalography forward calculation in realistic volume conductors. Phys Med Biol. 48, 3637–3652 (2003). 14680264 10.1088/0031-9155/48/22/002

[b53] Hipp J. F., Engel A. K. & Siegel M. Oscillatory synchronization in large-scale cortical networks predicts perception. Neuron 69, 387–396 (2011). 21262474 10.1016/j.neuron.2010.12.027

[b54] Benjamini Y. & Hochberg Y. Controlling the false discovery rate: a practical and powerful approach to multiple testing. Journal of the Royal Statistical Society 57, 289–300 (1995).

[b55] Eickhoff S. B. *et al.* A new SPM toolbox for combining probabilistic cytoarchitectonic maps and functional imaging data. Neuroimage 25, 1325–1335 (2005). 15850749 10.1016/j.neuroimage.2004.12.034

[b56] Mazziotta J. C., Toga A. W., Evans A., Fox P. & Lancaster J. A probabilistic atlas of the human brain: theory and rationale for its development. The International Consortium for Brain Mapping (ICBM). Neuroimage 2, 89–101 (1995). 9343592 10.1006/nimg.1995.1012

[b57] Mitra P. P. & Pesaran B. Analysis of dynamic brain imaging data. Biophys J 76, 691–708 (1999). 9929474 10.1016/S0006-3495(99)77236-X PMC1300074

[b58] Jarvis M. R. & Mitra P. P. Sampling properties of the spectrum and coherency of sequences of action potentials. Neural Comput. 13, 717–749 (2001). 11255566 10.1162/089976601300014312

[b59] Nichols T. E. & Holmes A. P. Nonparametric permutation tests for functional neuroimaging: a primer with examples. Hum Brain Mapp. 15, 1–25 (2002). 11747097 10.1002/hbm.1058 PMC6871862

[b60] Destrieux C., Fischl B., Dale A. & Halgren E. Automatic parcellation of human cortical gyri and sulci using standard anatomical nomenclature. Neuroimage 53, 1–15 (2010). 20547229 10.1016/j.neuroimage.2010.06.010 PMC2937159

[b61] Nolte G. *et al.* Identifying true brain interaction from EEG data using the imaginary part of coherency. Clin Neurophysiol. 115, 2292–2307 (2004). 15351371 10.1016/j.clinph.2004.04.029

